# Color outside the lines: How does amoral management influence employees’ creative unethicality?

**DOI:** 10.1371/journal.pone.0347530

**Published:** 2026-04-17

**Authors:** Lei Ren, Yan Liu, Abdul Waheed Siyal, Xiaobin Zhang, Tianxing Pu

**Affiliations:** 1 School of Digital Economics and Management, Wuxi University, Wuxi, China; 2 School of Business, Jiangnan University, Wuxi, China; 3 School of Business Administration, Henan University of Economics and Law, Zhengzhou, China; Zhejiang University, CHINA

## Abstract

Existing research has confirmed that amoral management can induce employees to engage in unethical behavior. However, its influence on a specific form of misconduct that involves creativity—namely, creative unethicality—remains unclear. Based on social information processing theory, this study constructs a theoretical model examining the influence of amoral management on employees’ creative unethicality. Through a three-stage investigation of 249 R&D employees from three intelligent manufacturing companies in eastern China, the results reveal that amoral management positively influences employee moral decoupling. Moral decoupling leads to creative unethicality under high job creativity requirements. Job creativity requirements moderates the indirect effect among amoral management, moral decoupling, and creative unethicality. Specifically, when jobs require high creativity, amoral management positively influences moral decoupling, thereby inducing creative unethicality. This study combines amoral management and creative unethicality, broadening the understanding of creative unethicality origins from the viewpoint of leaders’ moral conduct. It also provides empirical insights for organizations to manage creative unethicality through job stressors and leadership development.

## Introduction

In recent years, the business world has witnessed numerous ethical scandals. Luckin Coffee fabricated sales transactions through related parties and inflated revenue using complex business models such as coupon redemptions and large corporate purchases. Meanwhile, Tesla’s Autopilot and Full Self-Driving systems have faced regulatory investigations over allegations that the company concealed accident data related to these technologies. When questioned, Tesla merely attributed the incidents to “system” errors. These organizational behaviors have attracted scholarly attention. Mai et al. (2022) coined the term creative unethicality to describe such conduct, defining it as “novel and innovative behavior that violates widely held moral norms” [1]. Compared to general unethical behavior, creative unethicality exhibits the following characteristics. First, it is innovative and cunning. Rather than violating rules overtly, actors employ intelligence, creativity, and a deep understanding of the system to devise novel and unconventional methods to circumvent regulations. Such behavior often requires high levels of cognitive ability and attention to detail, such as meticulously identifying and exploiting institutional loopholes. Second, it is concealed and ambiguous. Creative unethical acts often operate in gray areas of rules. Actors deliberately avoid clear-cut violations by distorting the intent of policies, capitalizing on unregulated spaces, or creating complexity to obscure their true intentions. As a result, these behaviors are difficult to detect and categorize promptly—examples include creative data manipulation and the use of workarounds. Third, it is strategic and calculative. This type of behavior is highly rational and driven by cost-benefit analysis. Actors behave selectively—disclosing only partial information, providing details only when inquired, and leveraging only those loopholes that offer high benefits with low risks. Each step is carefully considered to minimize the possibility of accountability. Scholars have called for further research to uncover the antecedents and mechanisms underlying creative unethicality, in order to curb its spread within business organizations [2].

Given the relatively novel concept of creative unethicality and its inherently unethical nature [1], we conducted a literature review on its antecedents, underlying mechanisms, and boundary conditions by integrating research on unethical behavior. Regarding antecedents, in terms of employee characteristics, studies have shown that individuals high in Machiavellianism [3] are more likely to exhibit creative unethicality, whereas those with high honesty-humility [4] are less inclined to engage in unethical behaviors. In terms of leadership, empowering leadership [1] and entrepreneurial leadership [2] have been found to positively influence creative unethicality. Other leadership and organizational contextual factors, such as leader immorality encouragement [5] and workplace multitasking [6] also promote unethical behaviors among employees. Regarding mechanisms, in the domain of intrinsic motivation, existing research has identified the role of creative support [1] in influencing creative unethicality. In terms of perceptions and attitudes, emotional exhaustion [7], self-conscious moral emotions [8], work alienation [9], relative deprivation [10] have been shown to facilitate unethical behaviors. In the realm of analysis and judgment, moral justification [2] and moral disengagement [3] have been found to trigger employees’ creative unethicality. Other cognitive mechanisms leading to unethical behaviors include moral awareness [6] and moral knowledge [4]. Concerning boundary conditions, existing studies have examined the influence of work environment factors such as performance pressure [1], error tolerance [3], as well as individual cognitive factors like politics perception [2], on the development of creative unethicality. Other organizational contextual factors, including ethical leadership [4], supervisor’s bottom-line mentality [7], job demands [9], and ethics-oriented HRM systems [11], along with individual traits such as trait mindfulness [6], have been demonstrated to influence the formation of employees’ unethical behaviors.

However, current research still has the following limitations. First, while existing studies have examined the antecedents of creative unethicality from the perspective of creativity-stimulating environments [e.g., 1,2], few have explored its antecedents from an ethical standpoint. Given that creative unethicality is inherently unethical, it is closely linked to organizational ethical management [2]. Morally-related leadership can serve a demonstrative function, influencing employees’ decisions regarding unethical behavior [11]. Such leadership can also be cultivated through selection, training, and cultural initiatives [12]. Therefore, investigating morally-related leadership as an antecedent of creative unethicality holds both theoretical and practical value. Second, the mechanisms underlying the generation of creative unethicality require further refinement. Existing theoretical frameworks suggest that creative unethicality is accompanied by distortions in moral judgment, such as those triggered by moral disengagement [3] or rationalization [2]. However, this perspective fails to explain why some employees choose to violate ethical standards while maintaining their personal moral principles [13]. Overlooking this mechanism may undermine the effectiveness of organizational training aimed at preventing moral decline, as employees might engage in such behaviors without altering their own moral beliefs [14]. Identifying these underlying information processing mechanisms can help organizations develop specific and actionable ethical strategies to manage such behavior. Third, an employee’s intention to violate ethical standards does not necessarily lead to creative unethicality [1]. It is also essential to consider the extent of job-specific creativity demands, which helps provide a more complete picture of when employees are likely to engage in such conduct. Identifying this boundary condition can assist organizations in tailoring intervention strategies to manage creative unethicality according to the nature of different roles.

To address the aforementioned research gaps, this study investigates the issue from the following perspectives. First, it examines the impact of amoral management on creative unethicality. The formation of creative unethicality requires employees to possess divergent thinking and cognitive flexibility [1]. Such cognition is influenced by an ambiguous ethical environment [2], which enables employees to solve problems in unethical yet novel ways. However, ethical leadership demonstrates clear support for moral norms [15], resulting in relatively low ambiguity within the ethical climate. In contrast, unethical leadership directly instructs subordinates to solve problems through explicitly immoral means [12], which does not necessarily require behavioral innovation. Compared to both ethical and unethical leadership, we argue that amoral management may serve as an antecedent to creative unethicality. It refers to “a leader’s consistent failure to respond to issues that have ethical implications” [16]. Scholars suggest that amoral management is widespread, largely because the business world is filled with moral gray areas [17]. Many decisions are not simply matters of right or wrong but involve complex trade-offs between competing interests [18]. Ethical leadership requires navigating these gray zones with moral intentionality—a process that can be arduous and potentially inefficient [4]. Unethical leadership explicitly chooses options that benefit oneself at the expense of others [5], whereas amoral management disregards moral dimensions altogether and bases decisions solely on economic rationality [16]. This approach becomes a survival strategy for operating efficiently in complex environments, helping to avoid decision-making paralysis [19]. Research on this form of leadership remains underdeveloped. The influence of amoral management has been discussed since the late 20th century [e.g., 17], and subsequent research has further conceptualized and developed theoretical models around it [e.g., 18,19]. Nonetheless, empirical studies in this area have progressed slowly. It was not until Quade et al. (2022) developed a measurement scale for amoral management that empirical investigations began to accumulate. Recent findings indicate that amoral management can promote unethical behavior among subordinates or within organizations [e.g., 20,21]. This study proposes that amoral management fosters a morally ambiguous work environment, which not only diminishes employees’ moral attentiveness [16] but also grants them greater latitude for discretionary actions [22]. Therefore, this study explores how and when amoral management influences employees’ engagement in creative unethicality.

Next, this study analyzes the mechanism of amoral management and the boundary conditions that induce employees to engage in creative unethicality. According to social information processing theory, people attend to environmental cues because they provide necessary guidance to help individuals function effectively in social settings [23]. These cues assist in forming different cognitions, which in turn shape decision-making frameworks and guide appropriate behaviors [24]. When the environment focuses an individual’s attention on economically related benefits, it triggers instrumental cognition, promoting profit-supporting behaviors; conversely, when attention is directed towards ethics and morality, it triggers ethical cognition and corresponding decision-making frameworks [24]. Amoral management fails to direct employees’ attention towards ethics, potentially leading employees to decouple moral performance from performance evaluations [21], resulting in moral decoupling. Moral decoupling refers to a “moral reasoning process whereby individuals separate their perceptions of morality from their perceptions of performance” [13]. This study hypothesizes that amoral management triggers moral decoupling among employees, which provides a cognitive foundation for unethical behaviors. However, whether these behaviors manifest as creative unethicality also depends on the work environment in which employees operate [25]. Social information processing theory suggests that individuals not only acquire information from closely related others but are also influenced by signals from their environment [23]. This study introduces job creativity requirement as a boundary condition between moral decoupling and creative unethicality. Job creativity requirement refers to employees’ perceptions of organizational expectations or needs for them to generate work-related creative ideas [26]. High levels of job creativity requirements may exert pressure on employees to innovate [27], potentially leading to creative unethicality under the influence of moral decoupling.

**Fig 1:**
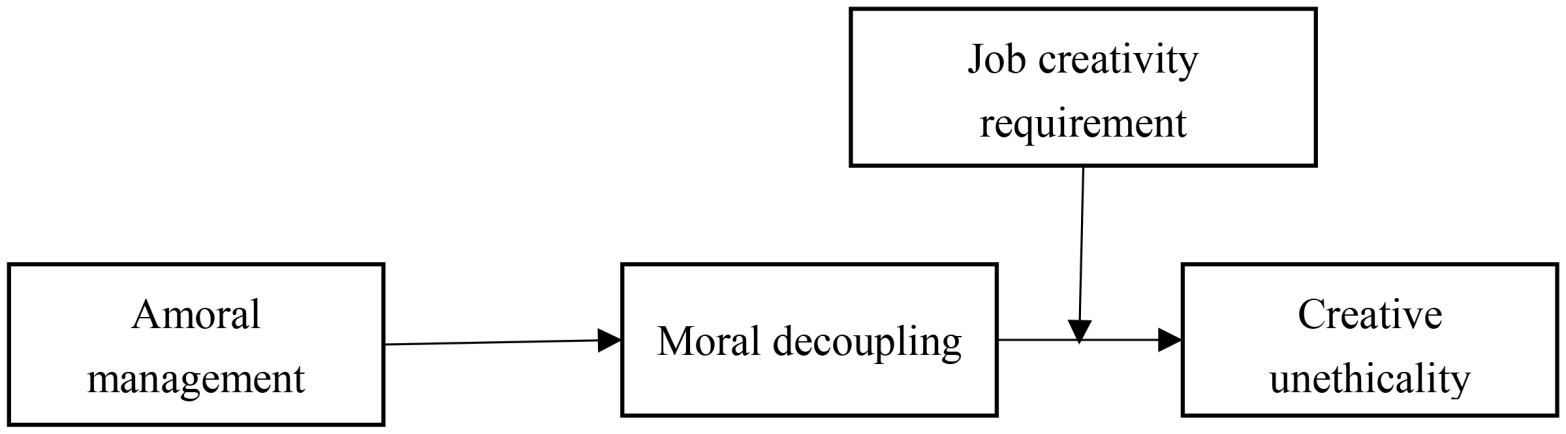
Theoretical model of amoral management and creative unethicality.

In summary, this study offers three key theoretical contributions. First, it advances research on creative unethicality by exploring amoral management as an antecedent—a novel perspective within the moral-related leadership domain. Simultaneously, it broadens the scope of outcomes associated with amoral management by demonstrating its influence on a specific form of unethical conduct. This finding corroborates the pervasive influence of amoral management in organizational settings. Second, this study uncovers moral decoupling as the mediating mechanism that explains how amoral management promotes creative unethicality in contexts with high job creativity requirements. Grounded in social information processing theory, we interpret this influence through the lens of signal perception, interpretation, and behavioral decision-making, thereby enriching the understanding of the mechanisms through which amoral management operates. Third, we identify job creativity requirement as a critical boundary condition. By demonstrating how high creativity demands strengthen the link between amoral management and creative unethicality, our research clarifies how leadership and job demands interact jointly to trigger creative unethicality, offering new insights into the contextual conditions that facilitate such behavior. The theoretical model of this study is illustrated in Fig 1.

## Theoretical foundation

### Amoral management

Carroll (1987) described amoral management as a form of leadership characterized by consistent inaction on ethical issues, whether intentional or unintentional, where leaders neither express preferences nor respond to decisions involving moral implications. Scholars have pointed out that amoral management differs distinctly from both ethical leadership and unethical leadership [18]. Ethical leaders not only adhere to moral principles in their daily lives but also demonstrate these values through personal actions and reinforce them via managerial processes such as communication and decision-making, thereby reflecting consistency in their individual and managerial moral character [28]. In contrast, leaders practicing amoral management often believe that rules in the business domain should be strictly separated from those in personal life, and they tend to avoid or remain neutral toward ethical issues [19]. Unethical leadership, on the other hand, involves actively deviating from moral standards and norms in decision-making, and may even employ unethical means to pursue personal or organizational interests [5]. Although amoral managers do not place significant emphasis on moral concerns, they do not proactively violate organizational norms or ethics, maintaining instead a neutral stance [19]. While this neutral attitude may appear non-disruptive on the surface, it can actually undermine the ethical foundation of an organization and encourage employees to engage in unethical behaviors [16], cheating behavior [22], and withdrawal [29].

### Moral decoupling

Moral decoupling is a distinct cognitive state pertaining to morality, initially identified through scholarly attempts to understand why consumers continue to support companies engaged in unethical practices [30]. This form of moral reasoning does not entail condoning unethical behavior; rather, it involves a psychological process that separates judgments of personal performance from ethical considerations [14]. Fehr et al. (2019) and Zhang et al. (2022) found that when leaders exhibit a high degree of moral decoupling, they tend to give higher performance evaluations to employees who engage in unethical behavior. Moral decoupling and moral disengagement are two distinct concepts within moral psychology. Although both involve cognitive processing related to unethical actions, they differ significantly in their core implications [22]. Moral disengagement refers to an individual’s use of cognitive strategies—such as moral justification, displacement of responsibility, distortion of consequences, or attribution of blame—to rationalize their own unethical behavior and avoid self-condemnation [31]. In essence, moral decoupling emphasizes a disconnect in evaluating external actors without altering one’s own moral standards [32], whereas moral disengagement focuses on self-justification and involves an internal compromise of moral principles [33]. Existing research has shown that moral decoupling can induce expediency [32] and workplace cheating [22].

### Creative unethicality

Scholars have investigated workplace unethical behavior through two primary lenses: general unethical behavior and specific unethical behaviors. General unethical behavior refers to employees’ violations of organizational rules and ethical standards in the workplace, such as tardiness, theft of property, or trafficking of confidential documents [34]. Specific unethical behaviors include time theft [35], workplace deviance [4], employee expediency [9], unethical pro-organizational behavior [36]. Based on financial fraud cases involving companies such as Enron and Luckin Coffee, Mai et al. (2022) and Zhao et al. (2025) examined a distinct type of unethical behavior—creative unethicality. They emphasized that such behavior not only breaches ethical norms but also involves elaborately designed, systematic deceptive practices. Subsequently, creative unethicality has garnered increasing attention from both academic and practical communities. Although existing research has adopted integrated perspectives and developed concepts such as creative deviance and unethical pro-organizational behavior, these constructs differ meaningfully from creative unethicality. Creative deviance describes employees’ disregard or violation of managerial directives to further develop or pursue innovative ideas [37]. It diverges from creative unethicality in several aspects. First, creative deviance is visible and often carried out openly by bypassing organizational review processes [38], whereas creative unethicality is characterized by invisibility and concealment. Second, creative deviance centers on employees’ persistence in advancing their own creative ideas [39], while creative unethicality focuses on employing inventive methods to engage in misconduct. Unethical pro-organizational behavior refers to actions that violate societal values, morals, or laws in order to benefit the organization [40]. A key distinction lies in the emphasis on creativity: creative unethicality entails diverse and covert methods, whereas unethical pro-organizational behavior often involves overt violations that are more easily detectable by those affected [31]. Furthermore, unethical pro-organizational behavior is explicitly motivated by the desire to benefit the organization [41], while creative unethicality does not presuppose any specific motivational source.

### Job creativity requirement

In the context of the VUCA era, innovation has become a critical pathway for organizations to gain a competitive advantage [42]. In response, organizations have incorporated job creativity requirements into work design, expecting employees to perform tasks that are complex and creative in nature [43]. On one hand, job creativity requirements represent organizational role expectations, signaling to employees that creativity is a desired behavior, thereby reducing role ambiguity and legitimizing innovative actions [44]. On the other hand, such requirements also imply that employees’ performance should demonstrate creativity [45]. In other words, high job creativity requirements closely link innovation to performance, encouraging employees to believe that generating and implementing innovative ideas will benefit their work [46]. These requirements prompt employees to proactively adapt to changes in job tasks and content, with the expectation that they employ creative thinking and innovative approaches to address complex problems that constrain organizational development [47]. Empirical findings regarding the impact of job creativity requirements are inconsistent. Some studies suggest that they can directly enhance creativity [47], stimulate creativity through creative process engagement [46]. However, Hon (2013) found job creativity requirement inhibited service performance due to increased work pressure. Kim et al. (2010) found that proactive personality fosters creativity only under the combined condition of high job creativity requirements and high supervisor support for creativity. Liu and Cheng (2025) argue that the relationship between job creativity requirements and creativity is complex and requires more nuanced, context-sensitive investigation.

### Social information processing theory

Social information processing theory, proposed by Salancik and Pfeffer (1978), serves as an important framework for explaining how employees form work attitudes. The core premise of this theory is that employees’ work attitudes are not solely determined by objective job characteristics or pre-existing individual needs, but are significantly influenced and shaped by their social context. First, social information processing theory emphasizes that individuals actively seek and rely on social information when forming work attitudes and articulating their needs. This includes opinions from colleagues and supervisors, as well as information about their own past behaviors. Particularly in ambiguous situations, individuals place greater weight on behavioral cues from others. Second, the processing of social information is affected by the salience and relevance of the information. Information that is most noticeable and accessible tends to have a stronger impact on attitude formation. Moreover, individuals often construct explanations for their own behaviors and attitudes that are perceived as reasonable and socially acceptable within their environmental context. Tenbrunsel and Smith-Crowe (2008) further refined social information processing theory by proposing a two-stage signal processing model. In the signal sending stage, the environment emits strong cues that influence decision-makers’ interpretation of the situation, directing their attention toward either an ethical or an instrumental decision-making framework. In the signal processing stage, the activated framework is applied in making action decisions.

## Hypotheses development

### Amoral management and moral decoupling

Based on social information processing theory, individuals seek and use cues from their social environment (including significant individuals and environmental characteristics) to interpret reality and form their own perspectives, attitudes, and motivations [23]. Since supervisors in the workplace are among the most important social influencers for employees [48], employees tend to interpret their supervisors’ behaviors as acceptable norms within the social environment and internalize them as their acting rules [49]. This study proposes that there is a positive correlation between amoral management and moral decoupling.

First, according to the two-stage signal processing model [24], amoral management influences employees’ moral awareness rather than instrumental concerns. We argue that the normative signals conveyed by amoral leaders contribute to employees’ moral decoupling. By excluding moral considerations from decision-making, amoral managers implicitly signal that ethical issues are irrelevant to organizational success [20]. As a result, employees weaken the perceived connection between moral standards and work outcomes, which may lead to a tendency toward moral decoupling. Second, leaders’ persistent neglect of ethics fosters an organizational climate in which moral considerations become invisible, resulting in a lack of ethical oversight [50]. Weak ethical governance fails to impose appropriate sanctions on misconduct, leading employees to increasingly believe that unethical acts are acceptable as long as they achieve success [51]. This cognitively isolates ethical judgments from outcome-based evaluations. Third, leaders serve as behavioral role models whose actions and attitudes cascade down to shape employees’ work behaviors [52]. Social information processing theory explains this process through schema alignment [23]. Amoral leaders view morality as inapplicable in business contexts [19]. Through both formal directives and informal leader-member exchanges [53], employees vicariously adopt leaders’ moral silence [21], ultimately perceiving ethical behavior as disconnected from performance metrics. Based on the above reasoning, this study proposes the following hypothesis:

Hypothesis 1: Amoral management positively influences moral decoupling.

### The moderating role of job creativity requirement on the relationship between moral decoupling and creative unethicality

Moral decoupling induces employees to engage in unethical behavior [13]. However, the emergence of creative unethicality hinges on organizations’ empowerment and expectations for creativity, making its genesis a more intricate process [1]. Social information processing theory emphasizes that individuals shape their attitudes and behaviors not only through social interactions with significant others but also by perceiving environmental features to make behavioral decisions [23]. During information processing, salient and relevant contexts can enhance members’ cognition and responses to signals [54]. Therefore, this study suggests that moral decoupling can only induce employees to engage in creative unethicality in specific contexts. This study hypothesizes that job creativity requirement may be a contextual factor catalyzing creative unethicality. Job creativity requirements refer to “participants felt that undertaking creative action was required for job performance” [55]. It conveys a clear message to employees: innovation is a valued and expected work behavior in the organization [44]. When employees perceive creativity as part of their job requirements, they are more likely to actively seek opportunities for innovation, try new methods and ideas [56]. Empirical research has found that job creativity requirements enhance employees’ service performance [26] and creativity [46]. This study argues that moral decoupling leads to creative unethicality in the context of high job creativity requirements; otherwise, its impact on creative unethicality is insignificant.

Under high job creativity requirements, moral decoupling may have a positive effect on creative unethicality. First, according to the social information processing theory [23], high creativity demands convey salient instrumental cues indicating that innovation is a fundamental job requirement, thereby activating employees’ instrumental focus. When employees lack moral restraint and face heightened pressure to be creative, they may prioritize innovation over ethical adherence [57]. Although creatively unethical behaviors are morally problematic, they may nonetheless help employees meet creativity expectations to some extent [1]. Consequently, morally decoupled employees may exclude ethical considerations from their work domain and seek innovative—yet unethical—ways to achieve task objectives. Second, high creativity requirements inherently imply that creative output is closely tied to job performance [43]. Moral decoupling allows employees to focus exclusively on outcomes rather than the means of achievement, thereby reducing cognitive distraction [14]. Especially in contexts with high creativity demands, employees can concentrate more resources on exploring novel methods to enhance innovative performance [47]. In other words, when creativity expectations are elevated, morally decoupled employees are more motivated to engage in ethically questionable behaviors to achieve high levels of innovation, thus triggering creative unethicality.

Conversely, when job creativity requirements are low, the impact of moral decoupling on creative unethicality may not be significant. On the one hand, this may be because in situations with lower creativity demands, employees experience relatively little pressure to innovate [26]. They are less compelled to resort to risky or unethical means to achieve innovative outcomes quickly [58]. Therefore, in such a low-pressure environment, even if moral decoupling enables employees to initiate unethical behavior, they lack the external job-related incentive to act unethically in creative ways. This behavioral conservatism reduces the likelihood of employees engaging in creative unethicality. Furthermore, creative unethicality often involves certain costs and risks [59], such as potential penalties upon discovery, damage to reputation, and obstacles to career advancement [60]. In contexts where job creativity requirements are low, even if employees were to take risks and engage in creative unethicality, such behavior is unlikely to receive organizational endorsement or support [27]. Consequently, employees have little motivation to venture into creative unethical practices. Thus, moral decoupling is less likely to lead to creative unethicality under low job creativity requirements. Based on this reasoning, the following hypothesis is proposed:

Hypothesis 2: Job creativity requirements moderate the impact of moral decoupling on creative unethicality. When job creativity requirements are high, moral decoupling has a positive impact on creative unethicality; conversely, the impact of moral decoupling on creative unethicality is not significant.

### The moderated-mediation role of job creativity requirement

Given that individuals process social information through a sequence of acquiring information, forming cognition, and behavioral response [23], this study, having inferred the positive influence of amoral management on employees’ moral decoupling, further proposes that job creativity requirement moderates the relationship between moral decoupling and creative unethicality, we propose a moderated-mediation effect among amoral management, moral decoupling and creative unethicality under job creativity requirement.

We propose that in contexts with high job creativity requirements, amoral management exerts a positive indirect effect on creative unethicality through moral decoupling. By avoiding ethical issues, amoral management signals that morality is irrelevant, weakens the organizational ethical climate, and may trigger subordinates’ imitation of their supervisors [16], thereby facilitating moral decoupling among employees. Moral decoupling attenuates the close link between unethical behavior and negative work evaluations [32]. This is particularly salient in environments with high job creativity requirements, where tasks explicitly pressure employees to seek innovative solutions and where innovation is closely tied to performance evaluations [46]. Under such conditions, moral decoupling—prompted by amoral management—may combine with external performance pressure to stimulate divergent thinking, leading employees to devise new ways of bypassing ethical standards [3]. Moreover, cognitive flexibility may lead individuals to perceive creative unethical behaviors as acceptable in the given context [1], thereby resulting in higher levels of creative unethicality.

However, when job creativity requirements are low, amoral management is unlikely to influence creative unethicality through moral decoupling. Although amoral management leads employees to disregard moral considerations in decision-making and engages them in moral decoupling, thereby encouraging unethical conduct [22], low creativity requirements do not compel employees to innovate or achieve innovative outcomes [45]. In the absence of external pressure or intrinsic motivation to adopt novel approaches [47], employees are less inclined to employ concealed or creative methods to circumvent ethical norms, thus reducing the likelihood of creative unethicality. Therefore, this study proposes the following hypothesis:

Hypothesis 3: Job creativity requirement moderates the effect of amoral management on creative unethicality through moral decoupling. Specifically, when job creativity requirements are high, amoral management positively influences creative unethicality through moral decoupling; conversely, when job creativity requirements are low, the effect of amoral management on creative unethicality through moral decoupling is insignificant.

## Research methodology

### Sample and data collection procedure

The survey participants of this study were R&D employees from three large-scale intelligent equipment manufacturing companies in eastern China. The performance of products, the effectiveness of algorithms, and the reliability of decisions all depend on the integrity and authenticity of data or information [61]. Moreover, the smart manufacturing industry is characterized by high technological barriers, involving deep integration of hardware, software, and multiple modules, which requires substantial R&D investment [62]. Once R&D efforts succeed, companies can easily capture market share and reap significant returns [63]. This industry dynamic drives firms to continuously pursue optimal solutions with greater speed and lower cost [64], leading to high levels of time pressure and competitive anxiety within organizations [65]. At the same time, the rapid iteration of big data, artificial intelligence, and robotics technologies in smart manufacturing has surpassed the scope of existing ethical frameworks and legal regulations [66]. As a result, ethical guidelines and policies in the industry often lag behind, remain vague, and may even contain gaps [67]. Furthermore, the smart manufacturing sector is highly competitive, placing strong emphasis on cost-effectiveness, technological breakthroughs, and time-to-market [68]. This extreme focus on performance metrics may inadvertently marginalize the importance of ethical compliance and other social values [69]. The intense competition coupled with ambiguous ethical norms creates a specific context that may induce creative yet unethical behaviors.

Initially, the researcher contacted the human resource managers of these three companies and requested their assistance in selecting survey samples and distributing questionnaires. With the help of the human resource managers, the researcher obtained employee rosters. A random sample of 350 employees working on R&D was selected, and then the researcher sent emails to the selected sample asking if they agreed to participate in the survey. In the emails, the researcher explained the purpose of the study and the questionnaire distribution process, and guaranteed that the questionnaire survey was completely anonymous, and that the questionnaire data would be kept confidential and only used for academic research. Each questionnaire was assigned a unique number, but the researcher clarified to the participants that the number was only used to match questionnaires collected at different stages. Ultimately, a total of 302 employees agreed to participate in this survey.

This study employed a three-stage survey design. This approach temporally separates the core research variables to reduce the influence of internal factors such as participants’ consistency motives and transient mood states when completing the questionnaires [5]. It allows participants to provide relatively independent evaluations of different variables at different time points [45], thereby mitigating common method bias [70]. The time interval between survey stages should strike a balance between two methodological considerations. On the one hand, it should provide a sufficient time window for psychological processes to unfold and stabilize after being influenced by the independent variable, allowing the mediating mechanism to develop adequately [4]. On the other hand, it should minimize sample attrition by avoiding excessively long gaps, thus enhancing the reliability and validity of the findings [7]. A two-week interval was selected as it effectively balances these theoretical and methodological requirements [71]. This duration has been commonly adopted in empirical studies examining the relationship among organizational contexts, employees’ psychological states, and behavioral outcomes [e.g., 13,32,72,73]. Accordingly, a two-week interval was implemented between each survey stage in this study.

The questionnaires in the first stage measured employees’ perceptions of amoral management and demographic variables. A total of 302 questionnaires were distributed, and after eliminating incomplete responses and questionnaires where all answers were the same or showed obvious selection patterns, 285 valid questionnaires were recovered. Two weeks later, the second-stage survey questionnaires were distributed to the valid samples recovered in the first stage, requiring participants to evaluate their moral decoupling and job creativity requirements over the past two weeks. Consistent with the questionnaire cleaning method used in the first stage, 267 valid questionnaires were recovered in the second phase. Two weeks later again, the researcher distributed a total of 267 third stage questionnaires to the respondents of the valid questionnaires recovered in the second stage, measuring participants’ creative unethicality, and 249 valid questionnaires were recovered, with an effective recovery rate of 93%. By sorting the questionnaires with the same codes together, all the survey data required for this study were obtained. The survey was conducted from October 10 to November 21, 2024.

Given that the measures of moral decoupling and creative unethicality in the second and third stages involve high ethical sensitivity, social desirability bias may threaten the validity of participants’ responses [74]. Social desirability bias can lead respondents to underreport unethical attitudes and behaviors, thereby attenuating the true relationships between variables and making it more difficult to detect hypothesized effects [75]. To mitigate the influence of social desirability bias, this study implemented several measures, including ensuring anonymity and confidentiality, using neutral wording, and maintaining time intervals between surveys [22]. Participants were explicitly assured that their responses were completely anonymous, that the data would be used solely for academic purposes, and that their participation would not affect their daily work. These steps were taken to reduce defensive reactions stemming from concerns about negative evaluation [76]. All items were phrased in neutral and non-judgmental language to avoid emotionally charged or morally loaded terms, thereby minimizing reactive distortion [77]. Furthermore, the two-week interval between survey stages helped reduce the pressure associated with reporting sensitive variables at a single time point [78].

The demographic data of the sample were shown in Table 1. Among the valid samples, males accounted for 62% of the total sample; the minimum age was 23, the maximum age was 57, and the average age was 34; most participants had bachelor’s degrees, with a total of 181 employees, accounting for 73% of the sample; 155 employees were ordinary staff, accounting for 62% of the total sample, and junior and middle managers together accounted for 33.7% of the total sample; 39% of the sample had been working for 4–6 years, followed by 7–10 years, accounting for 36%.

**Table 1:** Demographic indicators of the sample.

Indicators	Categories	Samples	Percentage
Gender	Male	154	61.85%
	Female	95	38.15%
Age	Below 25 years old	11	4.42%
	26-35 years old	173	69.48%
	36-45 years old	50	20.08%
	46-55 years old	8	3.21%
	Above 56 years old	7	2.81%
Education	Junior high school and below	0	0%
	High school or technical secondary school	9	3.60%
	Three-year college	14	5.60%
	University undergraduate	181	72.70%
	Graduate and above	45	18.10%
Position	Ordinary employee	155	62.20%
	Primary-level manager	52	20.88%
	Middle-level or department manager	32	12.82%
	Senior-level manager	10	4.10%
Tenure	Less than 1 year	4	1.61%
	1-3 years	18	7.23%
	4-6 years	96	38.55%
	7-10 years	90	36.14%
	More than 10 years	41	16.47%

### Measurements

The scales adopted in this research were all derived from mature Western scales, with the expressions of the Chinese versions determined through a translation-back-translation process [79]. A five-point Likert scale was employed, where 1 represents “completely disagree” and 5 represents “completely agree,” unless otherwise specified.

Amoral management. The four-item scale developed by Quade et al. (2022) was adopted. A representative item is “My supervisor does not get involved when ethical issues arise.” In this study, Cronbach’s α was 0.81.

Moral decoupling. The five-item scale developed by Fehr et al. (2019) was utilized. A representative item is “I believe that judgments of performance on work tasks should remain separate from judgments of morality.” In this study, Cronbach’s α was 0.78.

Job creativity requirement. The five-item scale developed by Yuan and Woodman (2010) was used. A representative item is “Introducing new ideas into the organization is part of my job.” In this study, Cronbach’s α was 0.77.

Creative unethicality. The six-item scale developed by Mai et al. (2022) was adopted, with a representative item being “Develops innovative ways to skirt ethical rules.” In this study, Cronbach’s α was 0.91.

Control variables. Consistent with the research by Zhao et al. (2025), this study controlled for employees’ gender, age, education, position, and tenure.

## Results

### Confirmatory factor analysis and common method bias test

We first examined the reliability and validity of the research variables. The factor loadings, composite reliability (CR), and average variance extracted (AVE) values for each variable were calculated, with the results presented in Table 2. The factor loadings for all variables were above 0.50, and the CR values were all above 0.70, indicating good reliability of the research variables [80]. Additionally, the AVE values of the research variables were greater than or close to 0.50, suggesting good convergent validity [81].

**Table 2:** Reliability and validity testing.

Constructs	Items	factor loading	α	CR	AVE
Amoral management	AM1	0.80	0.81	0.81	0.52
	AM2	0.75			
	AM3	0.71			
	AM4	0.62			
Moral decoupling	MD1	0.63	0.78	0.78	0.42
	MD2	0.66			
	MD3	0.67			
	MD4	0.72			
	MD5	0.56			
Job creativity requirement	IR1	0.69	0.77	0.77	0.40
	IR2	0.56			
	IR3	0.60			
	IR4	0.67			
	IR5	0.64			
Creative unethicality	CU1	0.80	0.91	0.91	0.64
	CU2	0.78			
	CU3	0.86			
	CU4	0.82			
	CU5	0.69			
	CU6	0.82			

By conducting confirmatory factor analysis using AMOS 21.0, we tested whether the variables in this study possessed discriminant validity, with the results presented in Table 3. As shown in Table 3, all fit indices of the four-factor model met the standard criteria (χ2/df = 1.80, CFI = 0.93, TLI = 0.92, RMSEA = 0.06). As shown in Table 3, the single-factor model exhibited the worst fit and did not meet the standards for various indicators, indicating that there was no severe common method bias in this study [82]. We also used Harman’s single-factor method to examine common method bias. The exploratory factor analysis results revealed that there were four factors with eigenvalues greater than 1, and the variance explained by the first factor was 21.60%, which was below the 50% threshold [70], further indicating that there was no significant common method bias in this study.

**Table 3:** Confirmatory factor analysis results.

Model	χ^2^	*df*	χ^2^/*df*	CFI	TLI	RMSEA
Four-factor model ^a^	295.22	164	1.80	0.93	0.92	0.06
Three-factor model ^b^	618.94	167	3.71	0.77	0.74	0.10
Two-factor model ^c^	888.56	169	5.26	0.63	0.59	0.13
One-factor model ^d^	1204.01	170	7.08	0.47	0.41	0.16

Notes: a Amoral management, moral decoupling, job creativity requirement, creative unethicality; b Amoral management, job creativity requirement, creative unethicality + moral decoupling; c Amoral management, job creativity requirement + creative unethicality + moral decoupling; d Amoral management + job creativity requirement + creative unethicality + moral decoupling.

### Descriptive statistics and correlation analysis

Descriptive statistics and correlation analysis were conducted on the study variables, with the results presented in Table 4. As shown in Table 4, amoral management was uncorrelated with creative unethicality (*r* = −0.01, *n.s.*). A positive correlation was observed between amoral management and moral decoupling (*r* = 0.35, *p* < 0.01). Additionally, moral decoupling was uncorrelated with creative unethicality (*r* = 0.11, *n.s.*).

**Table 4:** Descriptive statistics and correlation analysis.

Variables	1	2	3	4	5	6	7	8	9
1.Gender	1								
2.Age	−0.11	1							
3.Education	0.05	−0.05	1						
4.Position	0.02	0.07	−0.09	1					
5.Tenure	−0.12	0.66**	0.00	0.10	1				
6.Amoral management	−0.12	0.01	−0.05	−0.01	0.05	1			
7.Moral decoupling	−0.11	−0.08	0.07	−0.13*	−0.05	0.35**	1		
8.Job creativity requirement	−0.05	0.08	0.00	0.00	0.06	0.09	0.02	1	
9.Creative unethicality	0.05	0.02	−0.28**	−0.03	−0.01	−0.01	0.11	−0.06	1
*M*	/	34.09	/	/	/	1.83	1.70	4.40	2.23
*SD*	0.49	6.41	0.62	0.86	0.90	0.51	0.49	0.39	0.93

Notes: *p < 0.05, **p < 0.01.

### Hypothesis testing

The hypotheses were tested using hierarchical regression analysis in SPSS 21.0, with the results of the regression presented in Table 5.

**Table 5:** Hierarchical regression analysis.

	Moral decoupling		Creative unethicality		
Variables	Model 1	Model 2	Model 3	Model 4	Model 5
Gender	−0.24	−0.16	0.14	0.20	0.19
Age	−0.01	−0.01	0.01	0.01	0.01
Education	0.10	0.13	−0.46***	−0.48***	−0.49***
Position	−0.14	−0.13	−0.06	−0.04	−0.04
Tenure	−0.01	−0.03	−0.03	−0.04	−0.03
Amoral management		0.34***			−0.09
Moral decoupling				0.10	0.13
Job creativity requirement				−0.01	0.01
Moral decoupling × Job creativity requirement				0.28***	0.30***
*R* ^*2*^	0.04	0.15	0.10	0.15	0.16
Δ*R*^*2*^		0.12***		0.05*	0.01*
*F*	1.94	7.31***	5.14***	5.46***	5.04***

Notes: *p < 0.05, **p < 0.01, ***p < 0.001.

**Fig 2:**
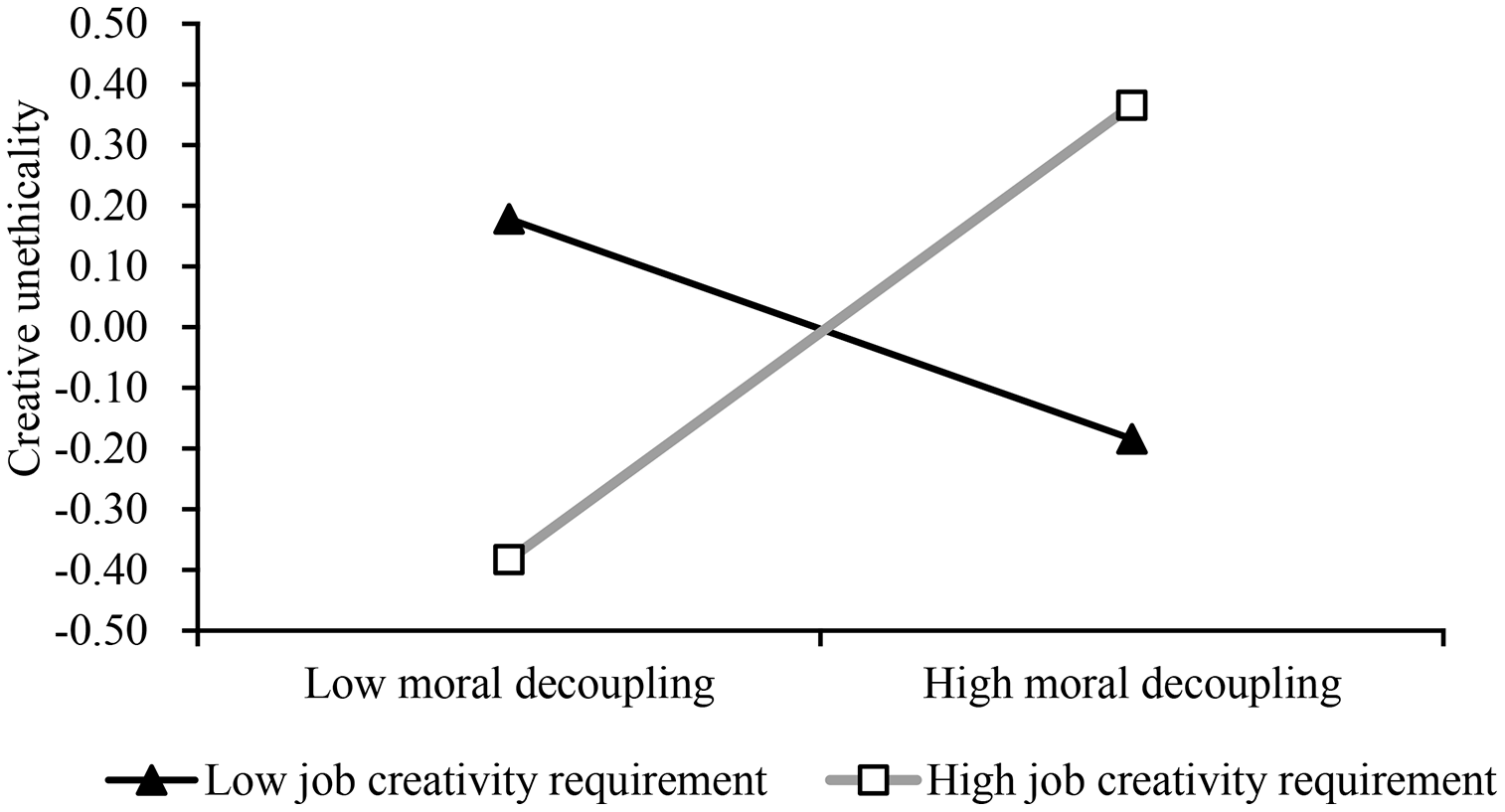
The moderating effect of job creativity requirement.

As shown in Table 5, amoral management positively influenced employee moral decoupling (*b* = 0.34, *p* < 0.001, Model 2), thus confirming Hypothesis 1. When taking creative unethicality as the dependent variable and including moral decoupling, job creativity requirement, and their interaction term in the model, the results revealed that the effect of moral decoupling on creative unethicality was not significant (*b* = 0.10, *n.s.*, Model 4). However, the interaction term between moral decoupling and job creativity requirement had a positive impact on creative unethicality (*b* = 0.28, *p* < 0.001, Model 4), indicating that job creativity requirement moderated the relationship between moral decoupling and creative unethicality. Following the approach of Aiken and West (1991), this study plotted a moderation simple slope graph, as shown in Fig 2. When job creativity requirement was high, moral decoupling positively influenced creative unethicality (*b* = 0.38, SE = 0.10, *p* < 0.001); whereas when job creativity requirement was low, the effect of moral decoupling on creative unethicality was not significant (*b* = −0.19, SE = 0.12, *n.s.*). Therefore, Hypothesis 2 was supported.

Using the PROCESS plugin (MODEL 14), we examined whether job creativity requirement moderated the influence of amoral management on creative unethicality through moral decoupling. The results were presented in Table 6. When job creativity requirement was high, amoral management had a positive impact on creative unethicality through moral decoupling. However, when job creativity requirement was low, the influence of amoral management on creative unethicality through moral decoupling was not significant. Upon testing, the difference in mediation effect across different levels of job creativity requirement was significant (*diff* = 0.21, SE = 0.09, Boot 95% CI = [0.05, 0.41]), and the moderated mediation effect was also significant (*effect* = 0.10, SE = 0.05, Boot 95% CI = [0.02, 0.20]). Therefore, Hypothesis 3 was supported.

**Table 6:** Moderated-mediation effect test.

		Effect	Boot SE	95% CI
Amoral management→ moral decoupling → creative unethicality				
Job creativity requirement	High	0.15	0.05	[0.05, 0.25]
	Low	−0.06	0.05	[-0.18, 0.02]
Index of moderated-mediation effect		0.10	0.05	[0.02, 0.20]

## Discussion

Drawing on social information processing theory, this study examined the impact of amoral management on employees’ creative unethicality. The research found that amoral management positively influenced employees’ moral decoupling. Job creativity requirement moderated the influence of moral decoupling on creative unethicality. Specifically, when job creativity requirement was high, moral decoupling had a positive impact on creative unethicality; whereas when job creativity requirement was low, the impact of moral decoupling on creative unethicality was not significant. Additionally, the study revealed that under conditions of high job creativity requirement, amoral management increased employees’ moral decoupling, leading to creative unethicality.

### Theoretical implications

Firstly, this study integrates amoral management with creative unethicality, thereby expanding the research on the antecedents of creative unethicality. Existing studies have identified empowering leadership [1] and entrepreneurial leadership [2] as antecedents of creative unethicality. These leadership styles inherently either provide innovation support or impose work-related pressure, thereby driving employees to engage in unethical conduct in pursuit of innovative outcomes. However, these studies overlook the fundamentally unethical nature of such behavior, and few have explored the antecedents of creative unethicality from a moral-related leadership perspective [3]. The emergence of creative unethicality requires divergent thinking and cognitive flexibility [1]. This study finds that the ambiguous ethical environment fostered by amoral leaders is a factor that induces employees to adopt innovative yet unethical approaches to problem-solving. By introducing amoral management, this study broadens the theoretical perspective and identifies a novel antecedent of this behavior. Furthermore, previous studies have primarily emphasized the dark side of amoral management, particularly its influence on employees’ general unethical behavior [16]. Researchers have suggested that future studies should pay more attention to how amoral management affects distinct forms of unethical behavior [22]. By introducing creative unethicality, this research enriches the literature on amoral management from a behavioral outcome perspective.

Secondly, this study identifies moral decoupling, specifically within contexts of high job creative requirements, as the underlying mechanism that links amoral management to creative unethicality. This refines the explanatory process through which amoral management shapes employee behavior. Previous studies based on moral conation theory have shown that amoral management suppresses moral courage among employees [16]. Incorporating social information processing theory, this research reveals that moral decoupling acts as a mediating mechanism between amoral management and creative unethicality under high creative work demands. These findings not only deepen the mechanistic insight into amoral management but also extend the application of social information processing theory. On the one hand, existing research on the emergence of unethical behavior has often emphasized how employees adjust their own moral cognition, highlighting the roles of moral disengagement [3] and moral justification [2]. Yet these studies have largely overlooked situations in which employees engage in unethical conduct without altering their moral standards—namely, through moral decoupling. The current study demonstrates that amoral management, characterized by stripping moral considerations from business decisions [16], implicitly encourages moral decoupling in employees, thereby complementing the mechanistic understanding of amoral management. Furthermore, this study validates the core pathway of social information processing theory: signal transmission, signal interpretation, and behavioral response [23]. It also supports the theory’s two-stage decision-making model. Amoral management initiates the development of employees’ moral cognitive frameworks and triggers moral decoupling [21]. Under high creative work demands, employees’ instrumental focus becomes activated, prompting instrumentally-driven decisions and resulting in creative unethicality. This process suggests that moral and instrumental cognitive frameworks are not entirely separate and may interact to shape employee decision-making under certain conditions.

Furthermore, this study explores the moderating role of job creativity requirement in the relationship between amoral management and creative unethicality, supplementing the boundary conditions of amoral management. Studies have found that organizational ethical climate exacerbates employees’ cognitive ambiguity regarding amoral management [16], while pay for performance strengthens the impact of amoral management on employees’ mental focus on work and self-interest cognition [21]. From the perspective of work stressors, this study further identifies job creativity requirement as a contextual factor influencing employees’ creative unethicality under amoral management. The findings indicate that amoral management itself conveys a signal of moral disregard to employees, and whether they engage in creative unethicality also depends on specific work situations. This study reveals that high job creativity requirement, combined with amoral management, tends to prompt employees to adopt covert and opportunistic means to engage in unethical behavior. Additionally, while prior research has found that job creativity requirement positively influences employees’ creativity [46], few studies have revealed its negative effects [83]. This study discovers that organizations’ emphasis on job creativity does not always yield positive outcomes. Especially in contexts where leaders adopt an amoral stance, the organization’s high expectations for job creativity among employees may instead lead to creative unethicality.

### Practical implications

This study offers the following practical implications for organizations. First, organizations should take proactive measures to constrain amoral management. On the one hand, it is recommended that organizations regularly conduct ethical leadership training. By establishing systematic curricula and analyzing typical ethical management cases, such training can deepen leaders’ ethical awareness and enhance their ethical decision-making abilities [22]. Organizational managers should be encouraged to actively participate in ongoing ethics education to enrich their knowledge of ethical theory and practice, thereby enabling them to proactively address ethical dilemmas [16]. On the other hand, organizations should improve their ethical management systems. This involves not only fostering an organizational culture that values ethics, but also clarifying institutional policies on ethical management [5]. Cultivating an ethical culture serves as a soft constraint, guiding managers to voluntarily adhere to ethical principles [4]. Meanwhile, formal ethical management systems provide clear guidance and a basis for leaders when making moral decisions [11]. These measures are designed to nurture a sense of moral responsibility among leaders and ensure that they uphold ethical standards when confronting complex managerial issues.

Second, organizations should actively identify and curb the emergence and spread of employees’ creative unethicality. Organizations can reduce such behavior by establishing ethics committees and conducting regular ethical assessments [3]. Ethics committees, characterized by independence and impartiality, are able to comprehensively monitor internal conduct and ensure that organizational members adhere to ethical guidelines. They also provide professional guidance and recommendations to employees [28]. When leaders remain silent on ethical issues, members can consult the ethics committee for advice to inform their behavioral decisions [20]. Furthermore, regular ethical assessments serve as another means to mitigate creative unethicality [4]. By developing detailed evaluation procedures and criteria, organizations can periodically review ethical practices, which helps identify and manage creative unethical conduct. These assessments also provide employees with a safe and reliable channel for reporting concerns and offering suggestions, thereby ensuring that ethical standards within the organization are effectively implemented and upheld [53].

Third, organizations should prudently manage employees’ creative job demands. For positions with high creative requirements, organizations can mitigate the emergence of creative unethicality by introducing ethical review mechanisms or establishing evaluation criteria that incorporate ethical dimensions of creative output [45]. The introduction of an ethical review process helps rigorously monitor compliance with ethical standards during project initiation and development phases. Halting ideas that violate ethical principles in a timely manner can reduce the likelihood that high creative demands pressure employees into taking shortcuts [46]. Additionally, organizations should develop evaluation standards that include ethical considerations. During the acceptance phase of creative projects, emphasis should be placed on the accuracy of data, adherence to regulations, and the reliability and integrity of information. Incorporating moral dimensions into project appraisal helps counteract the potential neglect of ethics due to creative job demands and guides employees toward responsible innovation [26].

### Limitations and future research

This study has the following limitations. In terms of research methodology, a three-stage survey approach was adopted, yet the conclusions cannot guarantee a causal relationship between the variables [84]. Future research should adopt more rigorous longitudinal data to provide stronger support for the mediating and moderating effects. The sample used in this study consists of R&D personnel from three intelligent equipment manufacturing companies. The geographical and professional characteristics of the sample limit the generalizability of the findings. Future studies could test the proposed model in other sectors, such as accounting and finance, by examining creative unethicality among professionals like accountants, auditors, and actuaries. The research context is based in China, where traditional cultural values such as *guanxi* [85] and power distance [86] may shape employees’ interpretations of amoral management. Further cross-cultural validation in Western contexts is needed to enhance the robustness and generalizability of the findings. Data were collected using self-reported questionnaires, which may introduce common method bias. However, both Harman’s single-factor test and the single-factor model fit indicated that common method bias was not a serious concern in this study [70]. Despite the practicality and academic acceptance of self-reports for measuring morally sensitive constructs like creative unethicality [3], this approach is susceptible to social desirability bias. Future research may obtain more objective measures through supervisor or peer ratings, or via archival records from human resources departments. Alternatively, experimental designs could be used to improve measurement validity.

In terms of research content, this study uses social information processing theory to explore the mechanism by which amoral management influences creative unethicality. Future research could integrate other theoretical frameworks to further refine the understanding of this mechanism. For instance, based on conservation of resources theory [87], researchers could explore the potential mediating role of ethical strain—defined as an individual’s experience of psychological strain in morally challenging situations [88]. Additionally, while this study focuses on the moderating role of job creativity requirements at the second stage, it does not investigate potential moderators at the first stage, such as variables that may influence the relationship between amoral management and moral decoupling. Future studies could also examine boundary conditions of amoral management by incorporating factors related to organizational human resource management (HRM) practices and individual characteristics. For example, ethics-oriented HRM practices [11] or employees’ moral identity [89] may attenuate the influence of amoral management on moral decoupling. These propositions warrant further empirical investigation.

## Supporting information

S1 File Supporting Information (S1_File.xlsx) to ensure transparency and replicability. (XLSX)
